# The value of cytokine levels in triage and risk prediction for women with persistent high-risk human papilloma virus infection of the cervix

**DOI:** 10.1186/s13027-019-0231-z

**Published:** 2019-06-28

**Authors:** Bohan Li, Ling Zhang, Jianguo Zhao, Guichun Tan, Wenwen Zhang, Na Zhang, Jing Tian, Pengpeng Qu

**Affiliations:** 1 Tianjin Central Hospital of Gynecology and Obstetrics, No. 156, Nankaisan Road, Nankai District, Tianjin, China; 2 Cang Zhou Central Hospital, No. 16 Xinhuaxi Road, Yunhe District, Cangzhou, Hebei China; 3 Tianjin Medical University, No. 22, Qixiangtai Road, Tianjin, Heping District China

**Keywords:** Cytokines, Flow cytometry, Cervical lesions, Triage, High-grade squamous intraepithelial lesion

## Abstract

**Background:**

Cervical cancer is a common cancer among women worldwide and is closely related to high-risk human papillomavirus infection (HR-HPV). The immune microenvironment is thought to play an essential role in viral infection and cancer development; however, this relationship remains controversial. Cytokines are an important part of the immune system. Therefore, in this study, we explored changes in cervical cytokine levels of women with persistent HR-HPV infection and determined the value of cytokine detection in assessing cervical lesions.

**Methods:**

We enrolled 146 patients; 117 had long-term high-risk (HR) HPV infection (> 6 months), and 29 were HPV-negative with previous HR-HPV infection. According to histopathological examination, 43 patients were diagnosed with cervicitis; 35, with low-grade squamous intraepithelial lesions (LSILs); and 39, with high-grade squamous intraepithelial lesions (HSILs). Cytokine levels in vaginal fluid were examined using cytometric bead array, and the values of interleukin (IL)-6 and IL-2 levels were converted to a cytokine score. The performance of the cytokine score for diagnosis and risk assessment was compared with that of ThinPrep cytology tests (TCTs).

**Results:**

Disease severity was positively associated with IL-6 levels and inversely related to IL-2 levels. The area under the curve (AUC) was higher for the cytokine score including IL-6 and IL-2 than for TCTs for HSILs. Comparisons of the sensitivity, specificity, Youden index, and positive and negative predictive values for HSILs demonstrated that the cytokine score was better than TCT. HPV-positive patients with high cytokine scores showed increased risk of developing HSIL within 3 years. The hazard ratio for the cytokine score was 3.12; thus, the risk of developing HSIL was related to the cytokine score.

**Conclusions:**

The cytokine score increased with the severity of cervical lesions and could distinguish more patients from HPV-positive women and predict the risk of disease progression.

## Background

Cervical cancer is the third most prevalent cancer in women, and most deaths occur in developing countries [1]. Epidemiological data combined with laboratory studies have identified persistent infection with high-risk (HR)-human papilloma virus (HPV) as a necessary condition for cervical intra-epithelial neoplasia (CIN) and cervical cancer [2]. Recently, with the development of HPV detection technology and improvements in screening strategies, clinicians have succeeded in increasing the prevention of cervical cancer [3, 4]. Currently, HPV detection has emerged as the primary screening method for cervical lesions in many countries [5, 6]. However, although HPV tests have high sensitivity, the specificity is poor. Therefore, it is necessary to assess the risk of cervical lesions and screen for abnormalities in patients with persistent HR-HPV infection.

Cytological examination combined with HR-HPV tests and evaluations in HPV-positive patients are the two most common methods for cervical screening today. However, persistent HR-HPV infection may not lead to cytological changes, and it takes time for HPV infection to develop into cervical lesions. Thus, interpretation of positive results of HPV tests is essential for guiding clinical practice and decision-making. Accordingly, clinicians are assessing the risks associated with monitoring cervical lesions in HR-HPV-positive patients. Triage and risk prediction based on local cytokines for HR-HPV infection can be used to screen for high-risk populations before cytological changes are detected. This method also relieves psychological stress while providing guidance for clinical practice.

The local immune microenvironment of the cervix also plays a crucial role in the clearance of HR-HPV [7]. Congenital immunity and acquired immunity have indispensable roles in this process. Therefore, HR-HPV immune escape is an important mechanism required for persistent HPV infection. Many researchers have reported that T cells play key roles in inhibiting persistent HPV infection [8, 9]. The immune response of lymphocyte populations induced by antigen stimulation can be reflected as changes in cytokine levels in the cervix. Th1 cells produce type I cytokines (e.g., interleukin [IL]-2, interferon [IFN]), whereas Th2 cells secrete type II cytokines (e.g., IL-4, IL-5, IL-6, IL-10, and IL-13) [10]. The Th1/Th2 imbalance is a key factor affecting the immune response in some pathological conditions and can lead to changes in cytokine levels. Therefore, cytokine profiles can reflect the local immune state of the cervix. Changes in some cytokines, such as IL-6 and IL-2, caused by persistent viral infection, can induce hyperplasia of the local vascular epithelium and activation of the Janus kinase (JAK)/signal transducer and activator of transcription (STAT) pathway, which result in local epithelial cell proliferation and disease progression [11].

In this study, we evaluated the application of cytokine detection in vaginal lavage fluid for patients positive for HR-HPV, and patients who did not develop high-grade squamous intraepithelial lesions (HSILs) were regularly followed up for 3 years [12]. We then compared the results of cytokine detection with those of cytological examination and assessed the use of cytokines for risk prediction.

## Materials and methods

### Patients

From August 2015 to June 2016, 146 patients who were treated at the Tianjin Central Hospital of Gynecology and Obstetrics were enrolled in this study after providing informed consent. Of these, 117 had long-term HR-HPV infection (> 6 months) and 29 were HPV-negative with previous HR-HPV infection. All patients underwent ThinPrep cytology tests (TCTs; threshold atypical squamous cells of undetermined significance [ASC-US]), and HPV was detected using the hybrid capture-II system. HPVs were genotyped by pyrosequencing. Vaginal lavage fluid was collected before cervical biopsy.

For the 117 patients eligible for this study, the inclusion criteria were as follows: (1) HR-HPV infection lasting longer than 6 months and (2) women aged 20–65 years. The exclusion criteria were as follows: (1) sex or vaginal medications within the last 3 days, (2) autoimmune diseases or immunodeficiency diseases, (3) cervical cancer or other malignant tumor diseases and serious medical conditions, (4) pregnancy; (5) supracervical hysterectomy, and (6) having taken drugs affecting immune function within the previous 6 months. For 29 HPV-negative patients, the inclusion criteria were the same as above except for HR-HPV infection.

### Study design

**Fig. 1:**
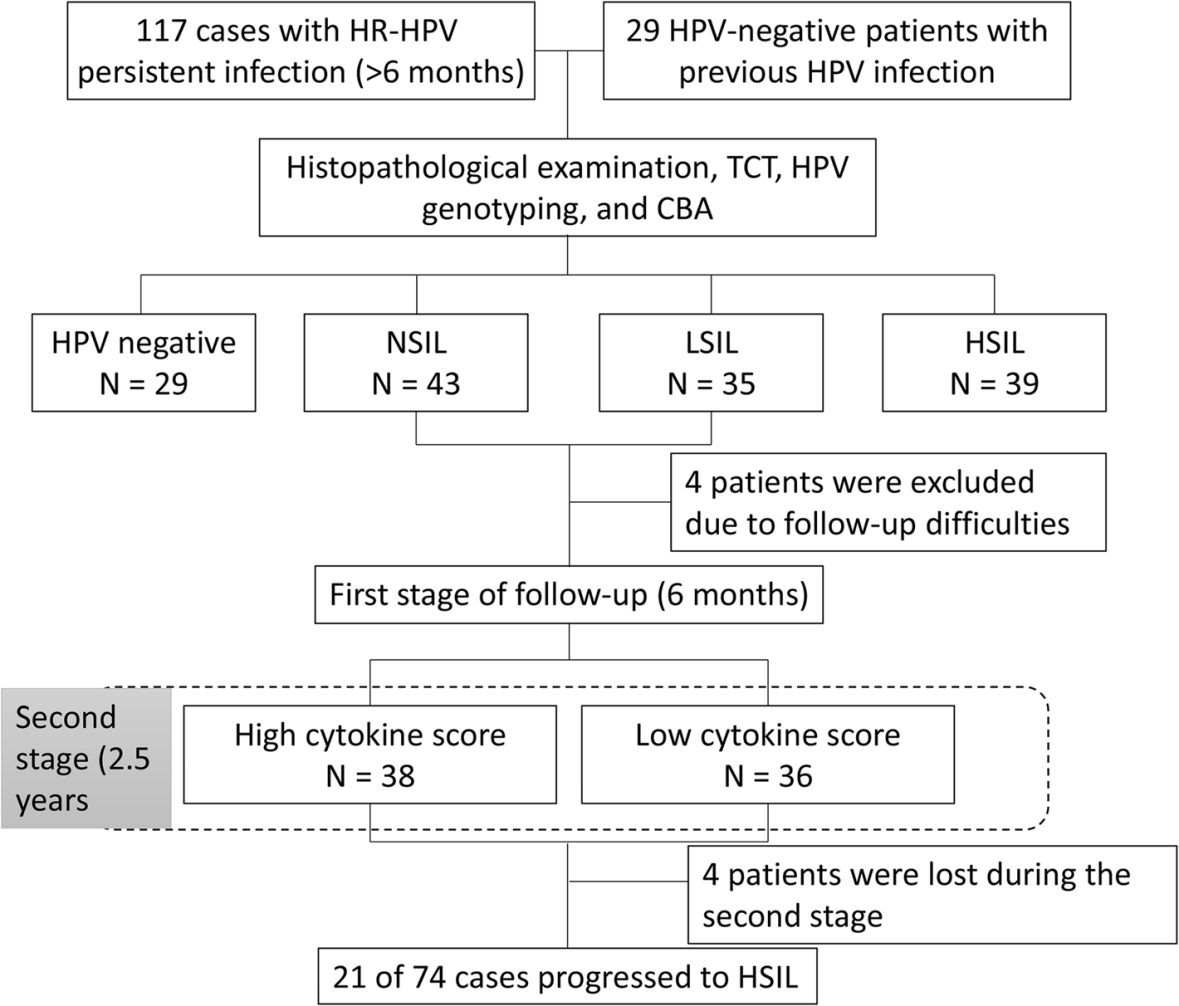
Flowchart of the study design

One hundred and seventeen patients with HR-HPV infection were divided according to the histopathological diagnosis as no cervical squamous intraepithelial lesion (NSIL; *n* = 43), low-grade squamous intraepithelial lesion (LSIL; *n* = 35), and HSIL (*n* = 39). There were no significant differences among groups with respect to age, HPV subtype, or pregnancy status. Seventy-four patients in the NSIL and LSIL groups were followed up for 3 years. The levels of IL-6, IL-2, IL-4, IL-17A, IL-10, tumor necrosis factor (TNF), and IFN-γ in vaginal lavage fluid were measured using a BD Cytometric Bead Array (CBA; BD Biosciences, San Jose, CA, USA) according to the manufacturer’s instructions. Statistically significant cytokines were converted to an algorithm as cytokine score, which was analyzed to be used as diagnostic panels by a logistic regression model. The follow-up was divided into two stages. The first stage was for 6 months; case data were collected and analyzed for the threshold of the cytokine score as a basis for grouping for the second stage (2.5 years) of follow-up. By comparing the results with TCT and HPV genotyping, a cohort study was conducted (Fig. 1).

### Specimen collection

Patients were positioned in the lithotomy position, and vaginal lavage fluid was collected before cervical biopsy. Five milliliters of sterilized physiological saline was used to wash the cervix and the upper one-third of the vagina. The saline was applied for 10 s, and then 4–5 mL of lavage fluid was aspirated from the posterior fornix. No blood component was permitted in the lavage fluid. The supernatant was collected after centrifugation (912×*g* for 5 min) and then stored at − 80 °C for cytokine measurement.

### DNA extraction and HPV genotyping

**Fig. 2:**
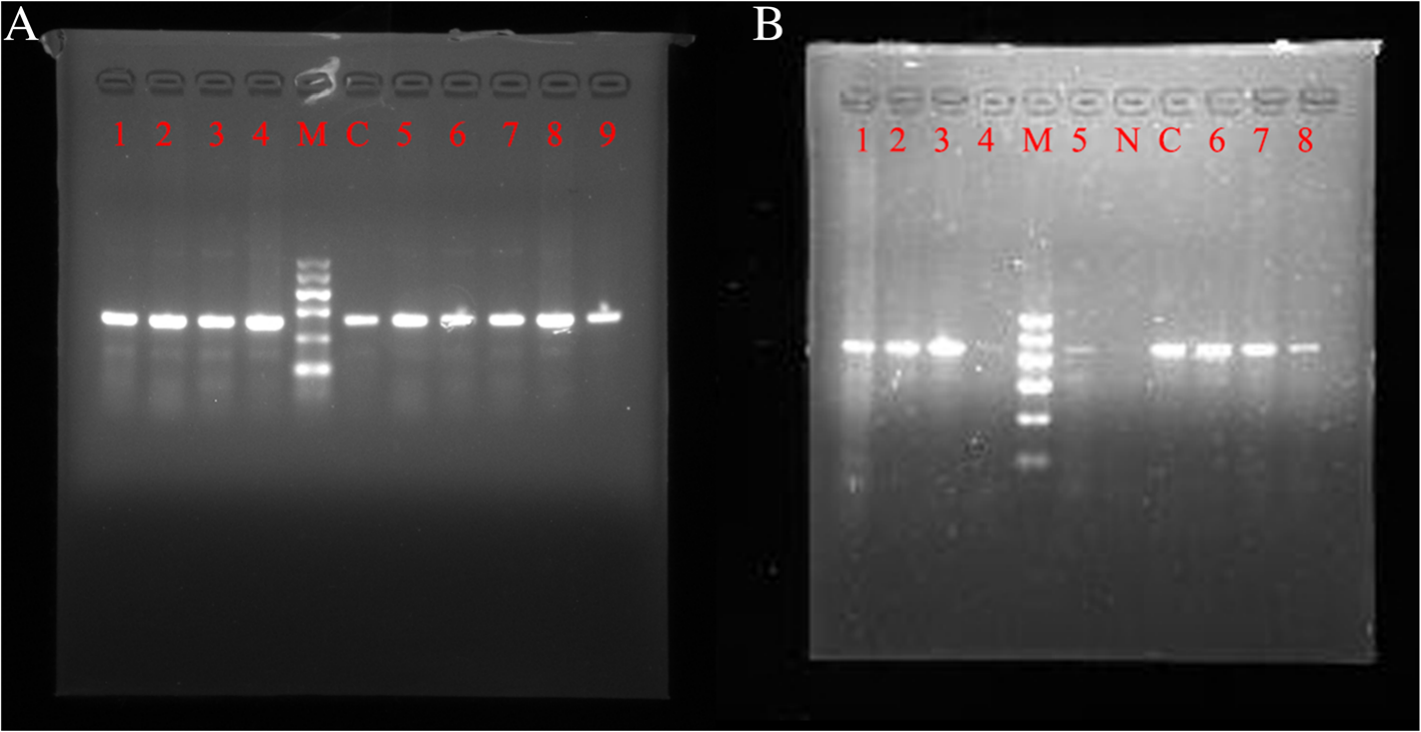
Gel electrophoresis of MY09/11 PCR (**a**) and GP5+/6+ PCR (**b**). M is Marker I, and C is the positive control (**a** and **b**). For (**a**), N is the negative control; weakly positive results were observed for samples 4 and 5, and others samples were positive. For (**b**), all samples were positive

**Fig. 3:**
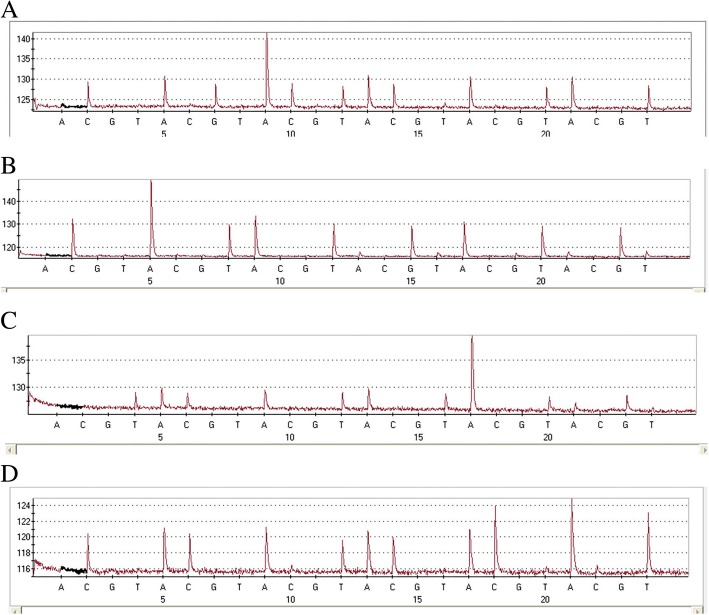
Different types of HPVs identified by pyrosequencing. **a**–**d**. Pyrosequencing could distinguish 20 HPV types, which were divided into four groups on average. HPV16 (**a**) was in group 1, HPV18 (**b**) was in group 2, HPV58 (**c**) was in group 3, and HPV6 (**d**) was in group 4

Exfoliated cervical cells were collected by a ThinPrep brush and then stored at 4 °C for DNA extraction, HPV detection, and genotyping. DNA was extracted using a protocol as described in the TIANamp Micro DNA Kit handbook (Tiangen Biotech, Beijing, China). DNA concentration was determined with a Nanodrop 2000 device (Thermo Scientific NanoDrop Products, Wilmington, DE, USA). DNA concentration was required to be higher than 50 ng/μL. DNA samples were stored at − 20 °C until use. HPV was detected by nested PCR using MY09/11and GP5+/6+ primer sets (Table 1, Fig 2) and pyrosequencing (Fig. 3) [13].

**Table 1:** PCR primer sequence and product length

Name	Primer sequence	Product length
MY09	CGTCCMARRGGAWACTGATC	450 bp
MY11	GCMCAGGGWCATAAYAATGG	
GP5+	TTTGTTACTGTGGTAGAACTAC	150 bp
GP6+	biotin-GAAAAATAAACTGTAAATCATATTC

### CBA

Cytokines in lavage fluid samples were measured with a BD CBA (Human Th1/Th2/Th17 Cytokine Kit; BD Biosciences) and a human transforming growth factor (TGF-β) enzyme-linked immunosorbent assay kit (IBL, Hamburg, Germany). The BD CBA kit was used for the simultaneous detection of human IL-2, IL-4, IL-6, IL-10, IFN-γ, TNF, and IL-17A. All protocols were performed according to the manufacturers’ instructions.

### Cytological evaluations

TCTs were performed according to the 2009 revision of the Bethesda system; 54 cases were negative for intraepithelial lesion or malignancy, 27 cases showed ASC-US, 11 cases showed atypical squamous cells without exclusion of high-grade lesions, 35 cases had LSILs, and 13 cases had HSILs.

### Histopathological examination

The histopathological diagnoses were made according to the World Health Organization criteria as follows: normal squamous cells with or without inflammation (normal or cervicitis), LSIL, or HSIL. From histopathological evaluation, 43 patients were diagnosed with cervicitis; 35, with LSIL; and 39, with HSIL. During the 3-year follow-up, 18 cases were diagnosed as developing into HSIL.

### Statistical analysis

Statistical significance was evaluated using SPSS22.0. Z tests were used to compare the area under the ROC curves. The cytokine levels among groups were analyzed by the t-test and Mann–Whitney U test. The correlation of IL-6 and IL-2 with the grade of cervical lesion was evaluated by Spearman’s correlation analysis. The logistic regression formulae, which included model intercept terms and coefficients for the IL-6 and IL-2 values, are as follows:

Predictive index (PI) for HR-HPV-positive women =2.588 + 0.259(IL6) − 1.582(IL2).

Calculation of cytokine score (%) $=\frac{e^{PI}}{1+{e}^{PI}}\times 100$.

The comparison between the cytokine score and TCT was analyzed by the McNemar test. The differences in the cytokine scores among groups were analyzed by the χ^2^ test. The Kaplan–Meier method was used to estimate the cumulative incidence (risk) of the histological outcome of HSIL after a 3-year follow-up. “Time to event” was defined as the number of years between the date of detection of abnormal cytokine levels and the date of histological diagnosis. HSIL was used as the endpoint in this analysis.

## Results

### Relative quantification of cytokine expression

**Fig. 4:**
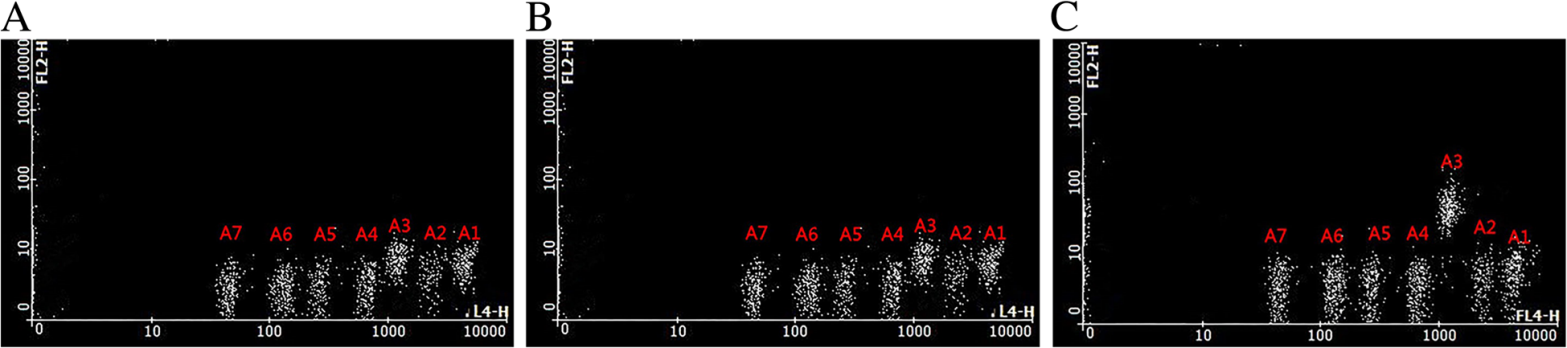
The BD Cytometric Bead Array for the detection of seven cytokines. **a**–**c**. Sample data from NSIL (**a**), LSIL (**b**), and HSIL (**c**). A1–A7 indicate IL-2, IL-4, IL-6, IL-10, TNF, IFN-γ, and IL-17A, respectively

We analyzed the relationships between cytokine levels (ng/L) and the degree of cervical lesion in the patients. The levels of IL-6 and IL-2 differed significantly among the three groups (NSIL/LSIL/HSIL; *P* < 0.05). These data showed that the level of IL-6 was lower for women with LSIL compared to those with HSIL (*P* = 0.00) and higher compared to those with NSIL (*P* = 0.00). However, the IL-2 level in LSIL was lower than that in NSIL (*P* = 0.00) and higher than that in HSIL (*P* = 0.058). Nevertheless, when the cytokine levels were analyzed between the HPV-negative and NSIL groups, there was no significant difference (*P* = 0.150 and *P* = 0.283, respectively). No statistically significant differences were found in the levels of other cytokines (*P* > 0.05; Fig. 4 and Table 2).

**Table 2:** Comparison of cytokine levels in cervical lesions of different grades

Cytokines	Groups	N	Cytokine levels (pg/mL)	*P* ^*^		
IL-6	HPV-negative	29	4.72 (4.22–6.57)	Reference		
	NSIL	43	6.22 (4.46–8.71)	*P* = 0.150	Reference	
	LSIL	35	16.68 (12.25–26.25)	*P* = 0.00	*P* = 0.00	Reference
	HSIL	39	38.26 (33.14–48.66)	*P* = 0.00	*P* = 0.00	*P* = 0.00
IL-2	HPV-negative	29	7.89 (7.29–8.38)	Reference		
	NSIL	43	7.47 (6.89–8.24)	*P* = 0.283	Reference	
	LSIL	35	6.70 (6.34–7.11)	*P* = 0.00	*P* = 0.00	Reference
	HSIL	39	6.52 (5.89–6.99)	*P* = 0.00	*P* = 0.00	*P* = 0.058
IL-4	HPV-negative	29	5.03 (4.48–5.87)	Reference		
	NSIL	43	5.71 (4.32–7.02)	*P* = 0.190	Reference	
	LSIL	35	5.06 (4.43–6.07)	*P* = 0.824	*P* = 0.197	Reference
	HSIL	39	5.27 (4.17–5.83)	*P* = 0.696	*P* = 0.153	*P* = 0.903
IL-10	HPV-negative	29	3.62 (3.24–5.94)	Reference		
	NSIL	43	3.55 (2.94–6.11)	*P* = 0.663	Reference	
	LSIL	35	5.40 (3.19–6.20)	*P* = 0.513	*P* = 0.298	Reference
	HSIL	39	5.19 (3.40–5.78)	*P* = 0.343	0.177	0.961
TNF	HPV-negative	29	4.81 (3.55–5.97)	Reference		
	NSIL	43	4.38 (3.57–6.61)	*P* = 0.836	Reference	
	LSIL	35	5.52 (3.32–6.72)	*P* = 0.925	*P* = 0.916	Reference
	HSIL	39	5.07 (3.73–6.38)	*P* = 0.891	*P* = 0.666	*P* = 0.927
IFN-γ	HPV-negative	29	3.91 (3.07–5.94)	Reference		
	NSIL	43	3.24 (2.71–6.11)	*P* = 0.323	Reference	
	LSIL	35	3.74 (2.93–6.16)	*P* = 0.903	*P* = 0.448	Reference
	HSIL	39	5.09 (3.17–5.78)	*P* = 0.780	*P* = 0.157	*P* = 0.697
IL-17A	HPV-negative	14	10.86 (9.75–13.60)	Reference		
	NSIL	19	10.74 (9.48–12.15)	*P* = 0.477	Reference	
	LSIL	18	11.32 (9.60–13.06)	*P* = 0.849	*P* = 0.378	Reference
	HSIL	18	12.09 (10.51–13.21)	*P* = 0.424	*P* = 0.057	*P* = 0.462
TGF-β	HPV-negative	29	193.82 (155.13–249.52)	Reference	0.40	0.51
	NSIL	37	193.8200 (178.95–259.63)	*P* = 0.329	Reference	
	LSIL	35	200.56 (165.14–267.75)	*P* = 0.422	*P* = 0.573	Reference
	HSIL	39	202.23 (157.16–288.76)	*P* = 0.435	*P* = 0.433	*P* = 0.685

* The differences in cytokine levels among groups were analyzed by the *t*-test and Mann–Whitney *U* test

### IL-6 and IL-2 levels were related to disease severity

**Fig. 5:**
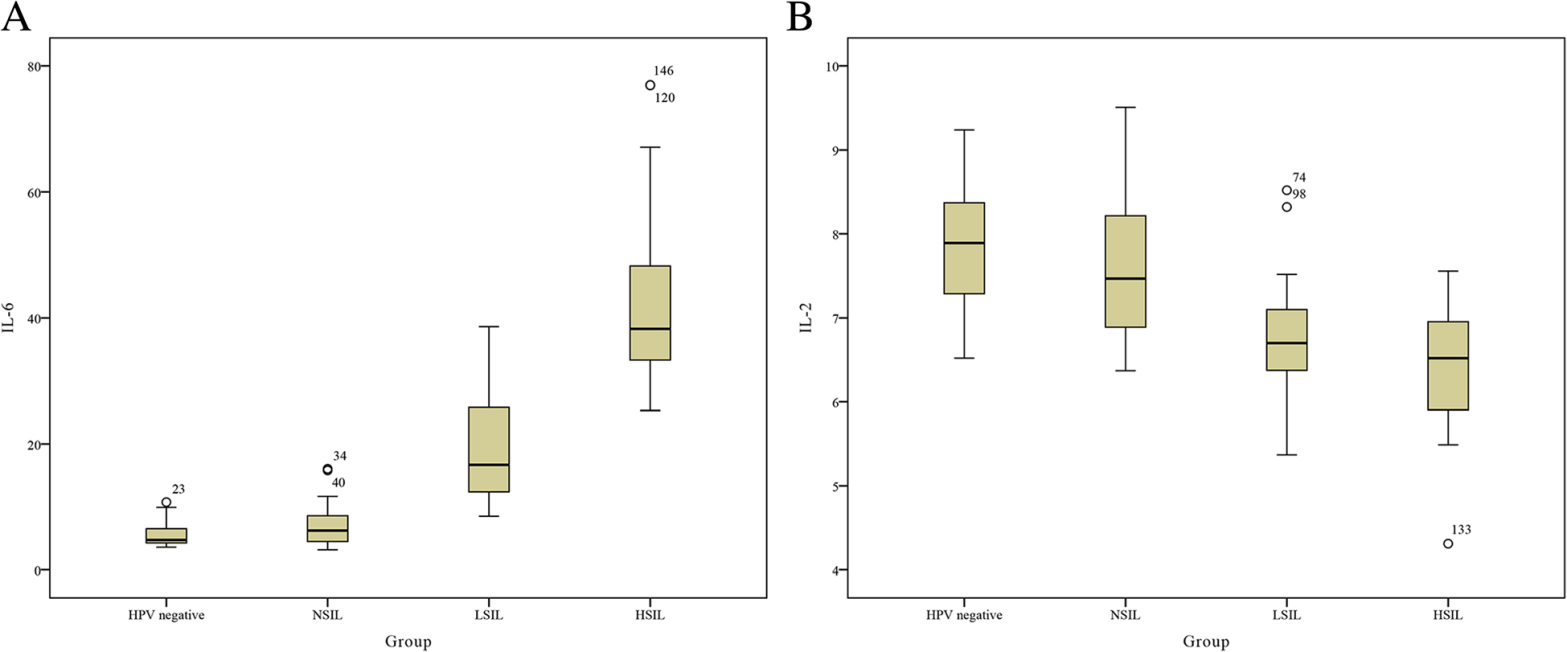
Levels of IL-6 and IL-2 in the HPV-negative, NSIL, LSIL, and HSIL groups. **a**, **b**. Sample data from IL-6 (**a**), IL-2 (**b**)

The levels of IL-6 increased with the severity of cervical lesions, whereas the opposite trend was observed for IL-2. Notably, there was a linear relationship between IL-6 and cervical lesions (R = 0.897). In contrast, the R value for IL-2 was − 0.551 (Fig. 5). Thus, these findings suggested that IL-6 and IL-2 were correlated with the progression of cervical lesions; accordingly, we chose IL-6 and IL-2 for further examination.

### Diagnostic scores for HR-HPV infection in women

After the analysis of cytokine levels, a second pilot study was completed, and logistic regression algorithms for HR-HPV-infected women were developed to diagnose HSILs. According to Table 3, IL-6 and IL-2 are independent risk factors for HSIL (*P* = 0.000 and *P* = 0.043, respectively). Because there was no significant difference in cytokine levels between HPV-negative and NSIL cases, the algorithms were developed using pooled data from HPV-positive patients (as mentioned in the Methods).

**Table 3:** Multivariate analysis of factors potentially influencing overall prognosis

Cytokine	B	OR (95% CI)	*P*
IL-6	0.259	1.30 (1.15–1.47)	0.000
IL-2	−1.582	0.21 (0.04–0.95)	0.043
Constant	2.588		

### ROC analysis and cut-off value

**Fig. 6:**
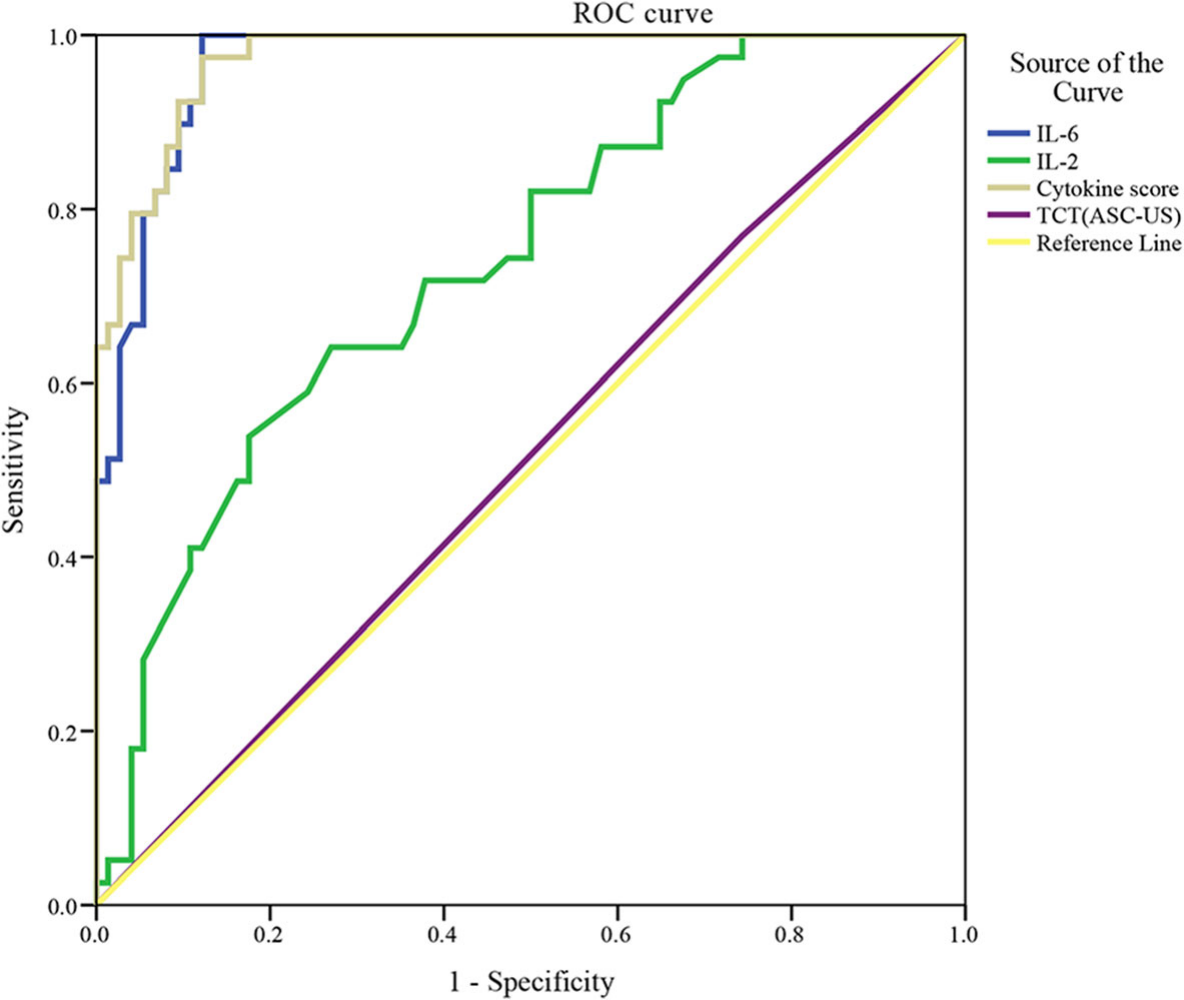
ROC curves of cytokine scores, IL-6, IL-2, and TCTs for HSILs

Compared with the values of IL-6, IL-2, and TCT, the ROC curve of the cytokine score was used to analyze its diagnostic efficiency. In the examination of HSIL, the AUCs of cytokine score, IL-6, IL-2, and TCTs were 0.974 (95% confidence interval [CI]: 0.952–0.996), 0.968 (95% CI: 0.941–0.995), 0.739 (95% CI: 0.645–0.833), and 0. 513 (95% CI: 0.401–0.625), respectively. As can be seen in Fig. 6, the AUC was the highest for cytokine score, followed by IL-6; TCTs showed the smallest AUC (*P* < 0.05).

### Efficacy of cytokine score and TCT for cervical lesion diagnosis

**Fig. 7:**
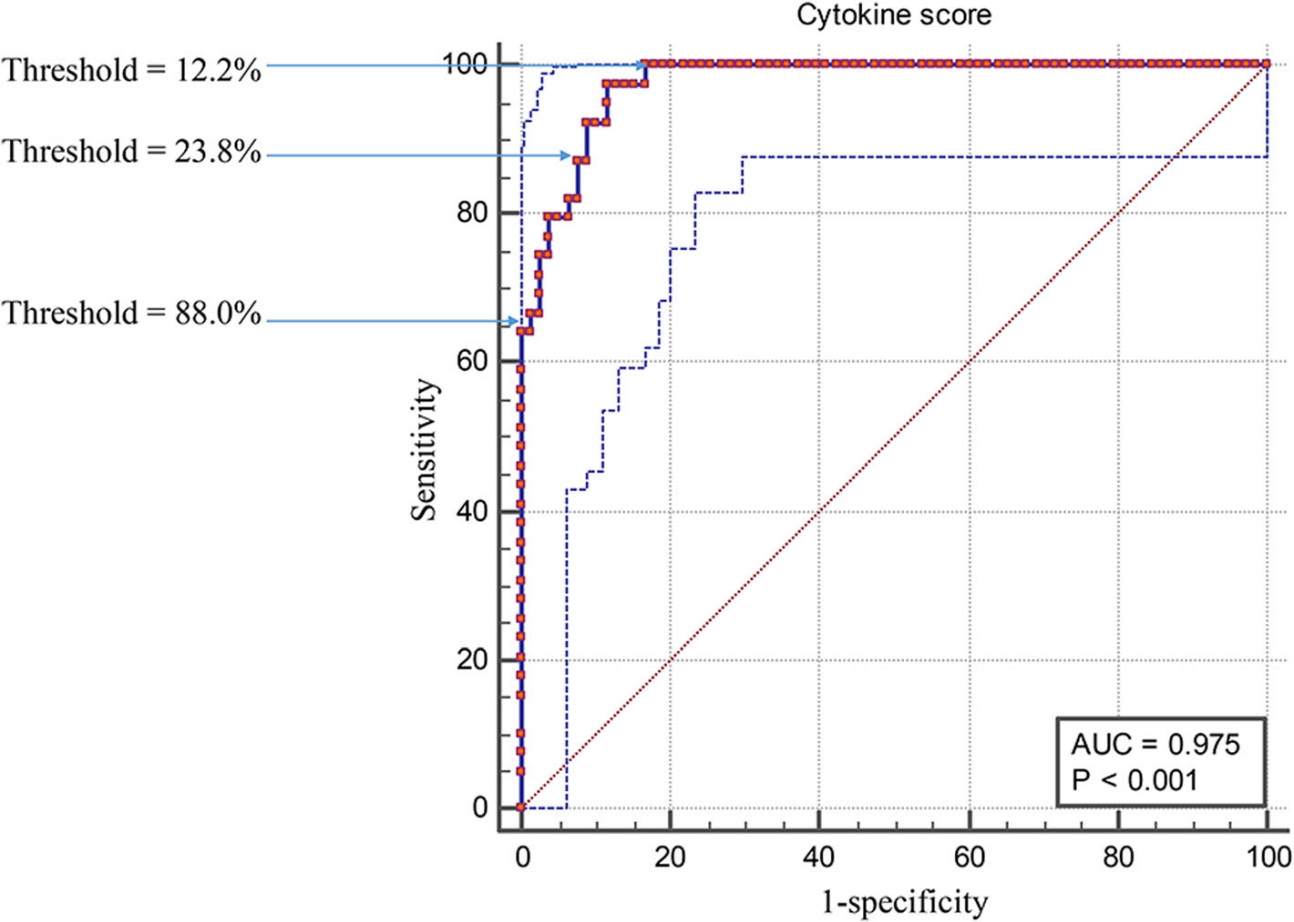
ROC curve analysis using different cytokine score cut-offs to detect HSILs. Dotted curves indicate the 95% CIs of the ROC of cytokine score (0.952–0.996)

The efficacies of the examination protocols were evaluated by comparing the sensitivity, specificity, Youden index (YI), positive predictive value (PPV), and negative predictive value (NPV). Moreover, according to the ROC curve of the cytokine score, sensitivities to detect HSIL at thresholds of 12.2, 23.8, and 88.0% were 100, 97.44, and 64.1%, respectively, and specificities to detect HSIL at thresholds of 12.2, 23.8, and 88.0% were 83.3, 88.5, and 100%, respectively (Fig. 7). The NPVs of the cytokine score increased simultaneously with lower cut-off values. The NPV of low and intermediate scores to detect HSIL were 100 and 98.6% respectively, which sharply declined to 84.8% in women with a high score (Table 4). In this study, we hoped to screen out as many HSIL patients as possible; therefore, 12.2% was chosen as the cutoff value.

**Table 4:** Comparison of the performance of cytokine score and TCT for cervical lesion triage

Threshold	Sensitivity	Specificity	YI	PPV	NPV
12.2%	100	83.3	83.3	75.0	100
23.8%	97.4	88.5	85.9	80.9	98.6
88.0%	64.1	100	64.1	100	84.8
TCT	76.9	25.7	0.03	34.1	69.0

The adjusted ORs for HSIL diagnosis in women by cytokine score and TCT were 383.30 and 1.15 respectively (*P* = 0.00) (Table 5). According to Table 3, for HSIL, the sensitivity, specificity, YI, PPV, and NPV of the cytokine score were superior to those of TCT.

**Table 5:** Effect of cytokine score and TCT on HSIL

Method	Threshold	Normal/LSIL	HSIL	OR (95% CI)	*P*
Cytokine score	< 12.2%	65	0	383.30 (22.17–6627.70)	*P* = 0.00
	> 12.2%	13	39		
TCT	<ASC-US	19	9	1.15 (0.46–2.86)	
	≥ASC-US	55	30		

### Follow-up of the first stage for HR-HPV-positive patients

Prior to the completion and analysis of the follow-up of the long-term incidence of HSIL presented in this paper, the first stage of follow-up was completed, and HR-HPV-positive women were divided into high-score and low-score groups according to the value of the cytokine score. To determine the threshold for long-term follow-up, the rates of progression to HSIL were compared for different cytokine scores over half a year. The thresholds were confirmed based on the formula for the cytokine score. According to Tables 6, 0.25% was chosen as the threshold for long-term follow-up.

**Table 6:** Comparison of the performance of cytokine score for risk prediction

Threshold (%)	Normal/LSIL	HSIL	OR	*P*
> 0.03	50 (79.4)	11 (100)	6.15 (0.34–111.13)	*P* = 0.097
> 0.25	29 (46.0)	9 (81.8)	5.28 (1.05–26.40)	*P* = 0.029
> 1.80	14 (22.2)	7 (63.6)	6.13 (1.56–23.97)	*P* = 0.005
> 11.92	10 (15.9)	4 (36.4)	3.03 (0.75–12.31)	*P* = 0.112

### Long-term incidence of HSIL in HPV-positive women according to baseline cytokine score and cytology results

**Fig. 8:**
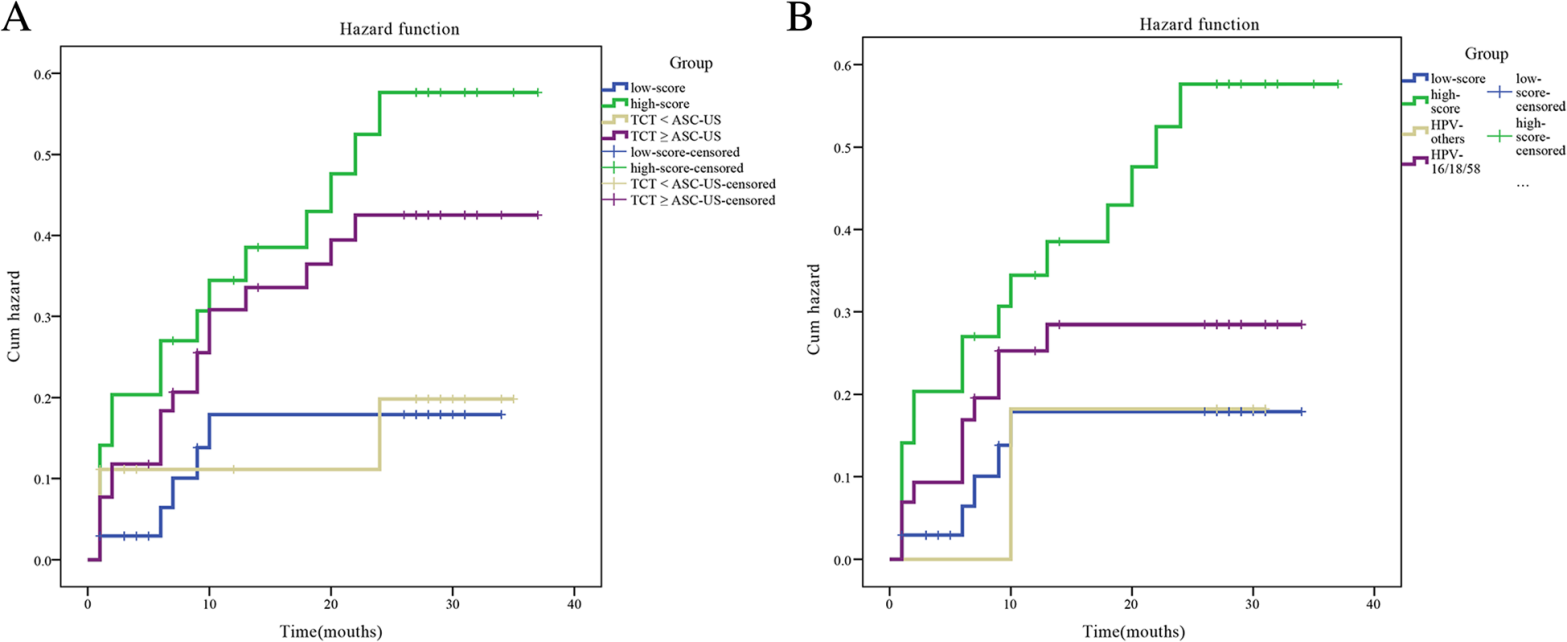
Three-year cumulative incidence of HSILs among HPV-positive women stratified by cytokine score, HPV genotyping, or TCT results at baseline. Kaplan-Meier incidence curves for HPV-positive women with high or low scores compared with HPV-positive women testing TCT < ASC-US or TCT ≥ ASC-US (**a**) and with a specific HPV subtype (**b**) at baseline

During the 3-year follow-up, 21 cases were diagnosed as developing into HSIL, and the results for HPV-positive women were stratified for cytokine scores. HPV genotyping, TCT, menopause, cytokine score, and age were analyzed for the hazard ratio (Table VII). HPV genotyping and TCT at baseline are depicted in Fig. 8. The cumulative incidence was 28.4% (95% CI: 20.1–36.6) after 3 years. The cumulative incidence of high-score patients was 42.1% (95% CI: 33.1–51.1), and those of HPV16/18/58-positive and TCT-positive patients were 23.0% (95% CI: 15.4–30.8) and 32.7% (95% CI: 24.2–41.3), respectively. The cumulative incidences of low-score and HPV16/18/58-negative and TCT-negative patients were 13.9% (95% CI: 7.6–20.2), 25% (95% CI: 17.1–32.9), and 15.8% (95% CI: 9.1–22.5), respectively (Table 7). Patients with a high cytokine score showed an increased risk of developing HSIL within 3 years (increased by 28.2%; 95% CI: 20.0–36.4). The HR for cytokine score was 3.12 (95% CI: 1.14–8.53), which was higher than those for TCT and HPV genotyping (1.98; 95% CI: 0.58–6.71 and 1.08; 95% CI: 0.29–3.97, respectively). Thus, the risk of developing HSIL was related to the value of the cytokine score as an independent risk factor. The median times to incidence for high-score and low-score patients were 24 (95% CI: 13–28) and 27 (95% CI: 26–29) months, respectively.

**Table 7:** Comparison of incidence rates

Factor	Status	Normal/LSIL	HSIL	HR (95% CI)	*P*
HPV subtype	HPV16/18/58	34	11	1.66 (95% CI: 0.21–12.86)	*P* = 0.628
	Others	5	1		
Menopause	Yes	13	3	0.54 (95% CI: 0.16–1.90)	*P* = 0.340
	No	36	14		
TCT	≥ASC-US	33	18	1.98 (95% CI: 0.58–6.71)	*P* = 0.275
	Normal	16	3		
Cytokine score	≥0.25	29	5	3.12 (95% CI: 1.14–8.53)	*P* = 0.026
	< 0.25	20	16		
Age (years)	≤50	36	14	0.55 (95% CI: 0.16–1.90)	*P* = 0.340
	> 50	13	3		

## Discussion

Numerous studies have assessed the biological and clinical relevance of cytokines, and they have been shown to be associated with the metastasis and progression of various cancers (including cervical cancer) [14, 15]. However, few studies have evaluated changes in cytokine levels and the clinical application of cytokine detection in the development of cervical lesions. Immunologically, patients with HPV infection lack specific CD4^+^ and CD8^+^ T cell reactions; thus, it is difficult to remove the virus [16, 17]. Moreover, changes in the Th1/Th2 balance might be involved in the pathogenesis of some diseases, including cervical lesions caused by HPV infection [10]. In a previous study, Th1-related cytokines in the cervix were found to be helpful for HR-HPV clearance; accordingly, patients with persistent infections tended to have decreased Th1 cytokines and increased Th2 cytokines.

Notably, changes in the levels of IL-6 also reflect the local immune status of the cervix [18]. However, activated lymphocytes, bone marrow cells, mononuclear macrophages, and some cancer cells (including cervical cancer cells) can produce IL-6, which is an important factor promoting the progression of the disease and the development of tumors. IL-6 has many important functions [19–21]. For example, with the development of disease, T cells display a shift in the Th1/Th2 balance, resulting in increased IL-6 secretion; this allows HPV-infected cells to evade the immune response of T cells. Moreover, IL-6 can induce the expression of vascular endothelial growth factor via the STAT3 pathway, leading to tumor angiogenesis and thereby promoting the development of cervical cancer. IL-6 has also been recently shown to have anti-apoptotic effects. In this mechanism, TGF-β inhibition is achieved by activating the phosphatidylinositol 3-kinase/AKT and JAK/STAT3 pathways, and recent studies have found that the IL-6/STAT3 pathway is closely related to cervical lesions and carcinogenesis caused by persistent HR-HPV infection; IL-6 and its receptors activate the JAK/STAT pathway, leading to high expression of the E6/E7 protein, which binds to and inactivates p53/pRb in HPV-infected cells [22, 23]. IL-2 can promote the apoptosis of diseased cells. In HPV-infected patients, the level of IL-2 in the peripheral blood of patients with cervical cancer is significantly lower than that in women without cervical lesions. IL-2 has antiviral and antitumor effects, and at high concentrations, it should be beneficial to the clearance of HPV infection and the prevention of lesion progression. Therefore, elevation of IL-6 levels is a risk factor for the development of persistent HPV infection, suggesting that IL-6 may have applications as an indicator of outcomes of HPV infection.

Helper T cells play an important role in the adaptive immune response, and various cytokines are involved in inflammation and carcinogenesis. IL-2, TNF, and IFN-γ are secreted by Th1 cells, which mediate immune responses associated with cytotoxic and local inflammation; therefore, Th1 is vital in anti-intracellular pathogen infections [24]. IL-4, IL-6, and IL-10 are secreted by Th2 cells, whose main functions are to stimulate the proliferation of B cells and produce immunoglobulin (Ig)-G and IgE. Th2-type cytokines are commonly seen during persistent infection with HR-HPV, and a previous study suggests that immunological markers can be used to predict the regression of HSILs [25]. Under normal circumstances, Th1 and Th2 cells are in a state of dynamic equilibrium, maintaining normal cellular immunity and humoral immune function. Persistent infection with HR-HPV promotes Th2 differentiation by downregulating Th1 cytokines and upregulating Th2 cytokines, and it can induce a shift from Th1 to Th2 dominance [26, 27].

In this study, we found that although most cytokines were not associated with HR-HPV persistence, increased IL-6 concentrations were closely related to disease severity. IL-6 is thought to be a pleiotropic cytokine that is closely related to inflammation and cancer. In this study, we found that the concentration of IL-6 in vaginal douche samples was higher in patients with more severe disease, consistent with histological findings and serum levels. Our findings thus suggested that increased IL-6 levels may be important for the persistence of HR-HPV infection and disease progression. However, further studies are needed to confirm these findings and elucidate the mechanisms mediating these effects. Furthermore, we also showed that the levels of IL-2, an antitumor cytokine, decreased as the severity of cervical lesions increased. It is possible that in response to a lack of IL-2, the host antitumor immunity decreased, resulting in disease progression. Accordingly, we can infer that decreasing IL-2 levels may result in the progression of cervical lesions. Again, additional studies are required to confirm this hypothesis.

Through the combined effects of IL-6 and TGF-β, CD4^+^ T cells differentiate into Th17 cells and secrete IL-17A, which plays a key role in host defense against infection. Previous studies have shown that persistent HPV16/18 infection and CIN are closely related to increased IL-17 levels; however, the role of IL-17 in viral persistence is still unknown. Under the influence of TGF-β, CD4^+^ T cells can differentiate into regulatory T cells (Tregs). Increased levels of cytokines, such as TGF-β, and elevated Treg numbers are commonly observed in HPV-related cancers. In our study, there were no differences in IL-17A or TGF-β levels among HPV-negative, NSIL, LSIL, and HSIL cases. This may be because the concentration was too low (e.g., close to the limit of detection of the assay). Alternatively, the lack of significance of the findings may be related to the small number of cases examined. Accordingly, additional studies with larger sample sizes are needed to confirm our findings.

The results showed that the AUC of cytokines for HSIL, especially the cytokine score, which includes IL-2 and IL-6, was significantly higher than that of TCTs, similar to the results for HSILs, suggesting that the cytokine score could be a good index for the identification of cervical precancerous lesions. In a comparison of the sensitivity, specificity, YI, PPV, and NPV of the two methods, with a cut-off of 12.2%, we showed that the cytokine score was superior to TCTs for HSILs. Thus, the cytokine score could identify more patients from HPV-positive women with a lower rate of missed diagnoses.

In this study, we also evaluated a cohort of HPV-positive women to determine the applicability of the cytokine score. In a test of the longitudinal sensitivity for HPV-positive women, we found that the risk of developing HSIL decreased with time and was higher in patients with high cytokine scores than in the general population, HPV16/18/58-type patients (in our previous study [13], we found that HPV58 was also highly prevalent in HSILs in Asia), and TCT-positive patients. We also showed that patients who were positive for HPV and had high cytokine scores had an increased risk of developing HSIL within 3 years; moreover, the risk of progression to HSIL was related to the measured value of the cytokine score, and patients with a high cytokine score developed HSILs faster than other patients. Overall, compared with TCTs and HPV genotyping, cytokine score detection was more valuable for risk prediction. In the clinical setting, women are currently tested every 3–5 years, and the threshold of 12.2% for HSIL detection can effectively prevent missed diagnoses. A threshold of 0.25% can indicate a higher risk of progressing to HSIL within 3 years. We showed that this triage method based on cytokine levels was objective and could be directly applied to most cases based on the HPV test, which has high repeatability and more advantages than TCTs.

The major strengths of the current study are its setting, i.e., nested within a clinical visit, and the formation of a study cohort after cervical examination. However, the sample size was small, and the follow-up time was relatively short. Accordingly, the number of patients who finally developed HSIL was low. Some patients were also lost to follow up, preventing us from accurately assessing the number of patients who progressed to HSIL. Furthermore, because this was not a multicenter study, the cytological examinations were somewhat subjective, and the diagnosis of TCTs might differ at other hospitals. Therefore, further multicenter studies are needed to confirm our findings. A previous study [28] showed that cytokine expression may be affected by bacterial vaginitis, trichomoniasis, and herpes virus infection. Because it was not possible to test all patients for these conditions, our study did not effectively eliminate all interference caused by other vaginal infections. Thus, additional testing is required.

## Conclusions

In summary, for HR-HPV-positive patients, cytokine detection with IL-6 and IL-2 was found to be superior to TCTs and HPV genotyping and was safe and objective. In addition, a high cytokine score may indicate a higher risk of developing HSIL.

## Data Availability

All data generated or analyzed during this study are included in this published article. [and its supplementary information files].

## Abbreviations

(IL)-6: Interleukin-6
AUC: Area under the curve
CBA: Cytometric Bead Array
CIN: Cervical intra-epithelial neoplasia
HR-HPV: High risk human papillomavirus infection
HSILs: High-grade squamous intraepithelial lesions
IFN: Interferon
JAK: Janus kinase
LSILs: low-grade squamous intraepithelial lesions
STAT: signal transducer and activator of transcription
TCTs: ThinPrep cytology tests
